# Mucosal injury during laparoscopic Heller cardiomyotomy: risk factors and impact on surgical outcomes

**DOI:** 10.1007/s00595-023-02680-2

**Published:** 2023-04-13

**Authors:** El-Sayed Abou El-Magd, Ahmed Elgeidie, Amr Abbas, Youssif Elmahdy, Ibrahem Lotfy Abulazm

**Affiliations:** 1 Department of General Surgery, Faculty of Medicine, Gastrointestinal Surgical Center GISC, Mansoura University, Gehan Street, Al Dakahlia Governorate, Mansoura, 35511 Egypt; 2 Faculty of Medicine, Mansoura University, Mansoura, Egypt

**Keywords:** Laparoscopic heller cardiomytomy, Mucosal injury, Outcomes, Risk factors, Pre-operative score for mucosal injury

## Abstract

**Purpose:**

To investigate the risk factors and outcomes of mucosal perforation (MP) during laparoscopic Heller myotomy (LHM) in patients with achalasia.

**Methods:**

We conducted a retrospective analysis of patients who underwent LHM for achalasia at a single facility.

**Results:**

Among 412 patients who underwent LHM for achalasia, MP was identified in 52 (12.6%). Old age, long disease duration, low albumin level, an esophageal transverse diameter  > 6 cm, and a sigmoid-shaped esophagus were found to be independent predictors of MP. These factors were assigned a pre-operative score to predict the perforation risk. MP had a significant impact on intra and post-operative outcomes. Gastric side perforation was associated with a higher incidence of reflux symptoms, whereas esophageal-side perforation had a higher incidence of residual dysphagia.

**Conclusions:**

Many risk factors for MP have been identified. Correctable parameters like low serum albumin should be resolved prior to surgery, while uncorrectable parameters like old age and a sigmoid-shaped esophagus should be managed by experienced surgeons in high-volume centers. Implementing these recommendations will help decrease the incidence and consequences of this serious complication.

## Introduction

Achalasia is a term used to describe a primary esophageal motility disorder, which usually manifests as dysphagia, chest pain, regurgitation, and weight loss [1]. Its etiology is still unclear, and it has a low annual incidence, ranging from one to three cases per 100,000 population [2]. The evaluation of a patient with achalasia patient entails clinical, radiographic, and endoscopic workup. However, a definite diagnosis is established only after manometric findings, which usually reveal absent esophageal peristalsis along with improper relaxation of the lower esophageal sphincter (LES) [3, 4].

The multiple treatment modalities for this disorder include medications (calcium channel blockers), endoscopic interventions (balloon dilatation, botulinum toxin injection, or peroral endoscopic myotomy POEM), and surgery (the Heller cardiomyotomy procedure) [4]. Heller described his initial experience of performing myotomy in 1913, in which he divided the anterior and posterior esophageal muscle fibers [5]. The surgical technique has been modified to include only division of the anterior muscle fibers, and it is now the standard myotomy procedure [6]. The main aim of surgical intervention is to divide the muscular fibers of the LES completely, on both the esophageal and gastric sides [7]. Delicate manipulation is required to preserve the mucosal membrane [8, 9]. As the mucosa is a thin layer that can be easily damaged, mucosal perforation could be encountered during this operation [10].

Since its establishment in 2002, Heller cardiomyotomy has been performed in our tertiary care center via open or laparoscopic approaches, and we have encountered cases of mucosal injury over these 18 years. The benefits, complications, and outcomes of this procedure have been documented; however, few trials have studied the predictors of accidental mucosal injury during this procedure or its impact on post-operative outcomes [11–13]. Thus, we conducted the current study to evaluate the risk factors for intra-operative mucosal injury after the Heller cardiomyotomy procedure performed for achalasia. We also assessed the impact of this complication on the intra- and post-operative outcomes of these patients.

### Patients and methods

This retrospective analysis was carried out at Mansoura University Gastrointestinal Surgical Center after approval from the Institutional Review Board of our medical school (IRB code: R.22.03.1642). The study was designed for patients with diagnosed esophageal achalasia, who underwent a laparoscopic Heller myotomy procedure between January, 2002 and December, 2020. After the exclusion of patients who underwent an open Heller myotomy procedure and those who had undergone a previous myotomy procedure and were scheduled for re-myotomy, 412 patients were the subjects of this analysis.

### Pre-operative assessment

All patients were asked about the four main symptoms of achalasia: dysphagia, regurgitation, chest pain, and weight loss. These symptoms were assessed via the Eckardt scoring system [14] and graded from 0 to 3, after which the total score was calculated and recorded. The severity of these symptoms was graded from 0 to 5 as follows: 0, absent; 1, mild; 2, moderate; 3, severe; and 5, very severe. The duration of symptoms was also recorded. Data on pre-existing medical co-morbidity or previous interventions for achalasia such as endoscopic balloon dilatation were collected. The patients’ condition was classified according to the American Society of Anesthesiologists (ASA physical classification) [15].

Clinical examination focused on appearance, body type, and body mass index (BMI). Routine pre-operative laboratory investigations, including serum albumin levels, were done for all patients. A barium meal was ordered for all patients to assess the esophageal shape and transverse diameter. The esophageal shape was classified as either straight or of the sigmoid type, whereas the degree of dilatation, according to the transverse diameter of the maximally dilated area, was graded from 1 to 3 as follows: Grade I, < 3.5 cm; Grade II, 3.5–6 cm; and Grade III, ≥ 6 cm [16].The esophageal shape was considered sigmoid if there was tortuosity or angulation in the lower esophageal segment; otherwise, it was considered straight [17]. Endoscopy was always performed to rule out malignant disease. Manometry was also done to assess the LES pressure, %LES relaxation, residual pressure, and the LES total and abdominal length.

### Laparoscopic Heller–Dor procedure

**Fig. 1:**
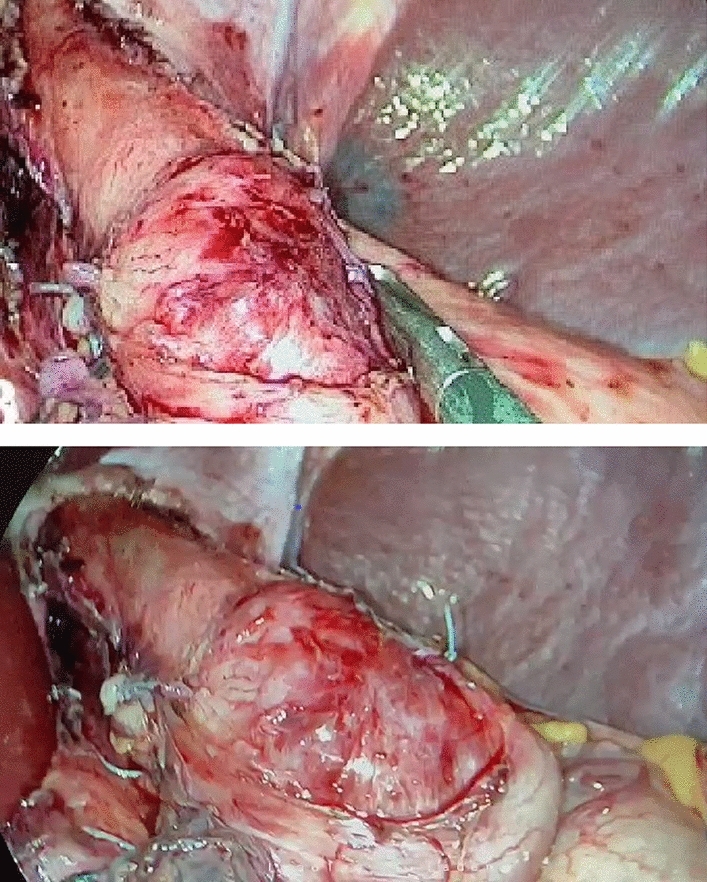
Completed myotomy

The laparoscopic Heller-Dor procedure was performed under general anesthesia with the patient in the French position. The camera port was inserted in the peri-umbilical region after abdominal insufflation. This was followed by the insertion of two working and two assistant ports. The lower abdominal esophagus was dissected from the phrenico-esophageal membrane and the two crura. After adequate exposure, a long myotomy of about 6 cm was performed on the esophageal side and 2–3 cm on the gastric side (Fig. 1). The myotomy was done via diathermy, with a harmonic scalpel (Ethicon Endo-Surgery, USA) or with a ligasure (Covidien, USA). Esophageal muscle thickness was classified as thin or thick based on the subjective evaluation of the surgeon. Then, the upper short gastric vessels were divided to prepare the fundus for Dor fundoplication, which was done using 2/0 silk sutures. A surgical drain was inserted under the left lobe, followed by a closure of the abdominal ports.

**Fig. 2:**
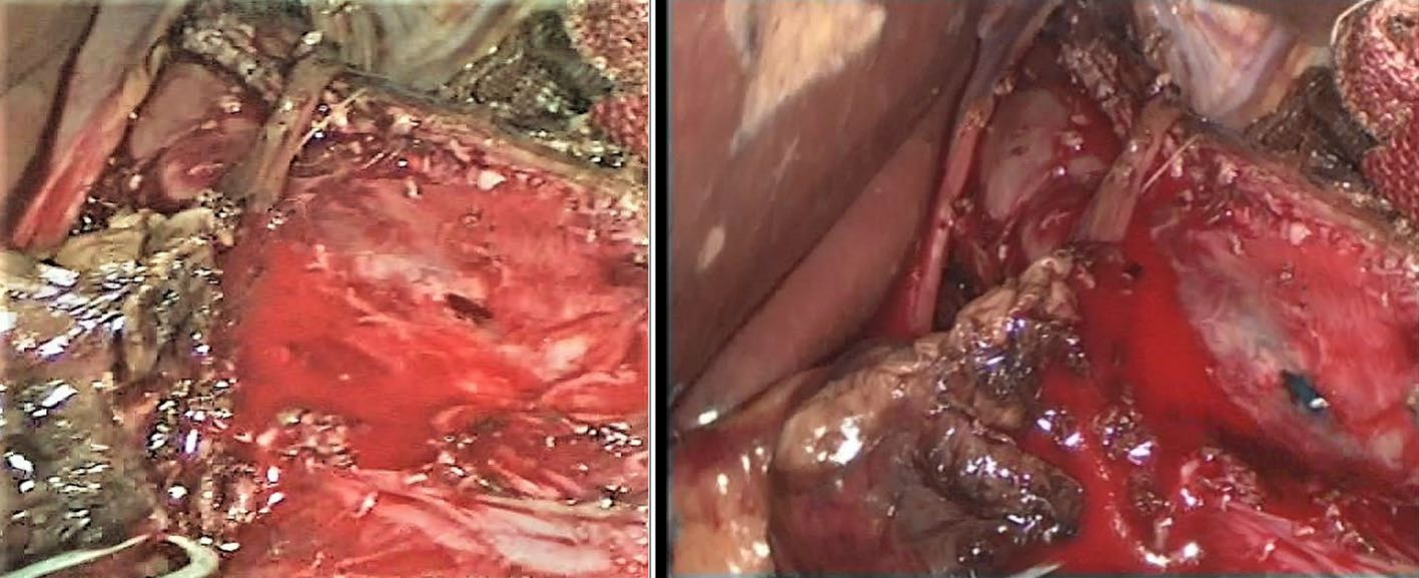
Esophageal mucosal perforation during laparoscopic Heller myotomy

**Fig. 3:**
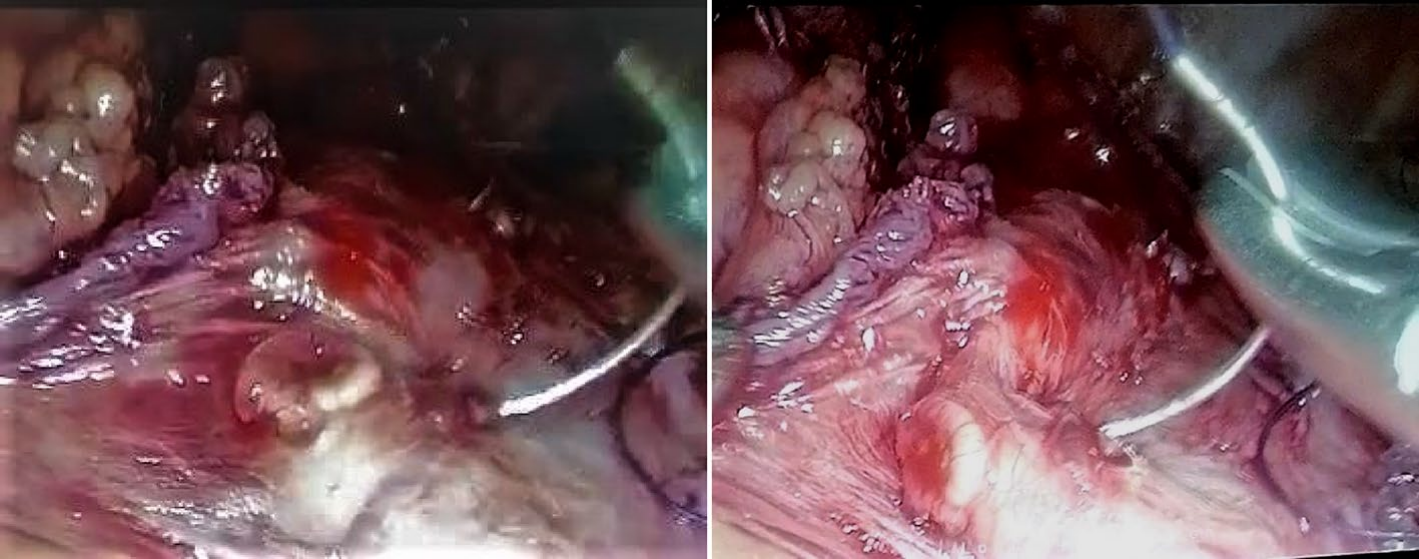
Gastric mucosal perforation during laparoscopic Heller myotomy

Mucosal injury was diagnosed when a full-thickness injury at the esophageal, gastric, or gastroesophageal junction was detected. It was repaired using interrupted 4/0 Vicryl sutures as in Figs. 2 and 3. Intra-operative blood loss and total operative time were recorded. The surgeon’s operative experience was graded from 1 to 4 as follows: < 5 cases, 5–10 cases, 10–15 cases, and more than 15 cases [11]. The rate of conversion to open surgery was also recorded.

### Post-operative evaluation and outcome

After the operation, patients were transferred to the recovery room and then to the ward for close monitoring. Most patients were allowed oral fluids on post-operative day 1 unless there were signs of complications. Any complications like fluid collection, delayed leakage, or the need for re-operation were recorded. Post-operative symptoms were assessed using the same Eckardt score. The total symptom score was calculated and a score of more than three was considered failure. Patient satisfaction with the surgical procedure was graded as very satisfied, satisfied, or unsatisfied.

### Follow up

Regular follow-up visits were scheduled for all patients. Patients were also asked about residual dysphagia and the need for its management by endoscopic balloon dilatation or by redo-heller myotomy. The development of GERD symptoms or reflux esophagitis according to the Los Angeles Classification was recorded. Patients were assigned to one of two groups according to the incidence of mucosal perforation: Group (A), patients with mucosal perforation; and Group (B), patients without perforation.

### Statistical analysis

The collected data were tabulated and analyzed using SPSS software for macOS. Normally distributed data are expressed as means and the standard deviation, whereas non-parametric data are expressed as median and the range. The independent samples t-test was used to compare the former data, whereas the Mann–Whitney U test was used for the latter. Categorical data are expressed as numbers and percentages. Groups were compared using chi-square or Fischer's exact tests. Multivariate regression analysis was used to assess the independent predictors of mucosal perforation. A p-value less than 0.05 was considered significant for all tests.

A scoring system was developed by correspondingly assigning weights to the risk factors based on the b coefficients from the final model, in which the points were estimated by taking the ratio of each risk factor’s β coefficient. Risk factors for mucosal injury were calculated using the Framingham study risk score functions. To classify the three risk groups, we used the estimated risk percentage of 10% and 50% as round cut-off values. The Brier score and the receiver operating characteristic curve were used for internal validation of the generated risk-scoring system.

## Results

### Patient characteristics

Based on our records, accidental mucosal injury was identified in 52 (12.6%) of the 412 patients who underwent laparoscopic Heller myotomy for achalasia. Group A comprised the 52 patients with mucosal perforation and group B comprised the remaining 360 patients who did not suffer this complication.

Older age was significantly associated with mucosal perforation, as the mean ages of the patients in Groups A and B were 49.1 years old and 41.1 years old, respectively (*p* < 0.001). However, gender did not have a significant impact on this complication. BMI showed a significant decline in association with mucosal perforation (21.38 kg/m^2^ vs. 24.32 kg/m^2^ in Groups A and B, respectively). The prevalence of smoking and other systemic co-morbidities did not differ significantly between the two groups (*p* > 0.05). The ASA scores were also similar. However, long disease duration was significantly associated with perforation (64 months vs. 24 months in Groups A and B, respectively). A history of endoscopic balloon dilatation was also more prevalent in Group A than in Group B (36.5% vs. 20.3%, respectively).

Analysis of the pre-operative symptoms revealed that the score and severity of dysphagia and chest pain were comparable in the two groups. However, the prevalence and score of weight loss were more prominent in Group A. Group A also had significantly higher scores and severity of regurgitation. The total pre-operative symptom score was significantly higher in Group A than in Group B (7 vs. 4, respectively; *p* < 0.001). (Table 1).

**Table 1:** Patient demographics and symptoms in the two groups

	Group A (*n* = 52)	Group B (*n* = 360)	*P* value
Age (years)	49.06 ± 15.78	41.11 ± 12.96	< 0.001*
Gender			
Male	24 (46.2%)	160 (44.4%)	0.817
Female	28 (53.8%)	200 (55.6%)	
BMI (kg/m^2^)	21.38 ± 3.75	24.32 ± 3.73	< 0.001*
Smoking	7 (13.5%)	45 (12.5%)	0.845
Neurological disease	0 (0%)	4 (1.1%)	0.445
Chest diseases	8 (15.4%)	33 (9.2%)	0.161
CVS diseases	5 (9.6%)	29 (8.1%)	0.702
DM	3 (5.8%)	11 (3.1%)	0.313
Metabolic diseases	3 (5.8%)	20 (5.6%)	0.950
ASA class			
I	38 (73.1%)	270 (75%)	0.744
II	13 (25%)	87 (24.2%)	
III	1 (1.9%)	3 (0.8%)	
Disease duration (months)	64 (12–120)	24 (2–120)	< 0.001*
Previous balloon dilatation	19 (36.5%)	73 (20.3%)	0.008*
Dysphagia score	3 (2–3)	3 (2–3)	0.079
Dysphagia severity			
Mild	1 (1.9%)	8 (2.2%)	0.084
Moderate	25 (48.1%)	229 (63.6%)	
Severe	26 (50%)	123 (34.2%)	
Chest pain score	0 (0–3)	0 (0–3)	0.667
Chest pain severity			
Absent	40 (76.9%)	286 (79.4%)	0.655
Mild	1 (1.9%)	9 (2.5%)	
Moderate	7 (13.5%)	51 (14.2%)	
Severe	4 (7.7%)	14 (3.9%)	
Regurgitation score	2 (0–3)	1 (0–3)	0.004*
Regurgitation severity			
Absent	16 (30.8%)	177 (49.2%)	0.008*
Mild	13 (25%)	100 (27.8%)	
Moderate	22 (42.3%)	81 (22.5%)	
Severe	1 (1.9%)	2 (0.6%)	
Weight loss	43 (82.7%)	106 (29.7%)	< 0.001*
Weight loss score			
< 5	1 (1.9%)	42 (11.7%)	< 0.001*
5–10	7 (13.5%)	36 (10%)	
> 10	35 (67.3%)	28 (7.8%)	
Total pre-operative symptoms score	7 (3–11)	4 (2–11)	< 0.001*

**Fig. 4:**
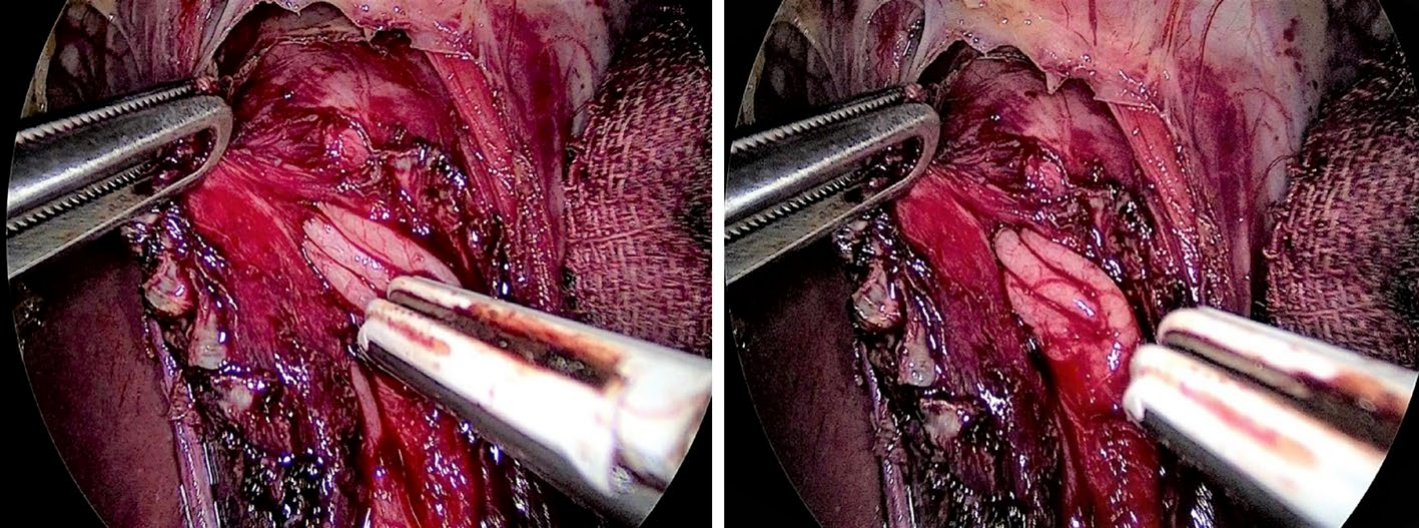
Redundant esophageal mucosa during laparoscopic Heller myotomy in a patient with sigmoid esophagus

Pre-operative serum albumin was significantly lower in Group A than in Group B (3.89 gm/dl vs. 4.03 gm/dl, respectively; *p* < 0.001). A sigmoid-shaped esophagus as shown in Fig. 4 was more common in Group A than in Group B (67.3% vs. 20.3%, respectively; *p* < 0.001). The degree of esophageal dilatation and its transverse diameter were also significantly greater in Group A.

Pre-operative manometric findings showed a significantly higher LES pressure but a significantly smaller LES total and abdominal length in Group A than in Group B. The residual pressure and % of LES relaxation were comparable between the two study groups. (Table 2).

**Table 2:** Pre-operative laboratory, radiological, and manometric data in the study groups

	Group A (*n* = 52)	Group B (*n* = 360)	*P* value
Serum albumin (gm/dl)	3.89 ± 0.29	4.03 ± 0.15	< 0.001*
Barium achalasia			
Straight type	17 (32.7%)	287 (79.7%)	< 0.001*
Sigmoid type	35 (67.3%)	73 (20.3%)	
Barium (degree of dilatation)			
< 3.5 cm	17 (32.7%)	227 (63.1%)	< 0.001*
3.5–6 cm	0 (0%)	62 (17.2%)	
≥ 6 cm	35 (67.3%)	71 (19.7%)	
Maximum transverse diameter (cm)	6 (2–8)	2 (2–8)	< 0.001*
Manometry LESP	48.17 ± 13.73	42.05 ± 12.53	0.002*
Residual pressure	11.85 (1–37.7)	4.5 (0–35.3)	0.813
Abdominal LES length	2.47 ± 0.51	3 ± 0.46	< 0.001*
Total LES length	3.59 ± 0.61	3.86 ± 0.50	0.001*
% LES relaxation	63.28 ± 14.16	63.21 ± 13.91	0.973

### Surgical outcomes of the patients with vs. those without mucosal injury

**Fig. 5:**
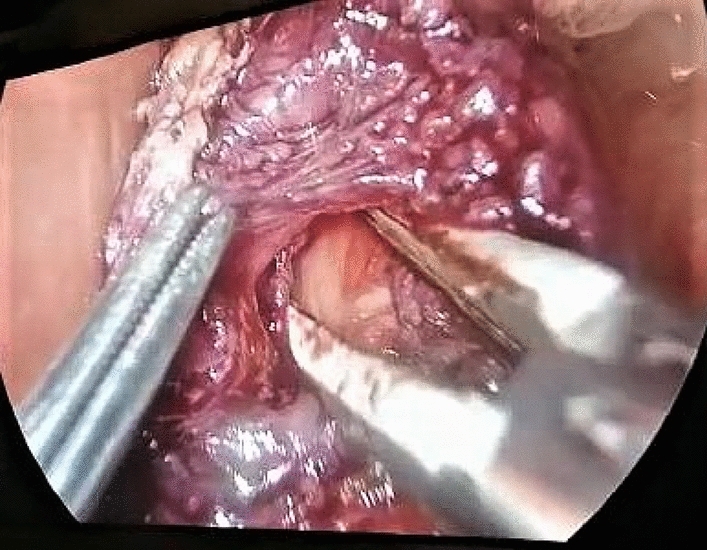
Thick muscle layer (deep plan of dissection)

Both blood loss and operative time were significantly higher in Group A than in Group B (*p* < 0.001). The degree of muscle thickness as shown in Fig. 5 (thin or thick) was comparable in the two groups. Limited operative experience carried some risk for mucosal perforation, as surgeons who had performed less than five LHM procedures in their career performed 26.9% and 7.5% of the procedures in Groups A and B, respectively. The harmonic scalpel was the most common method of dissection used, in 59.6% and 60% of the procedures in Groups A and B, respectively.

The occurrence of perforation was associated with a significant risk of conversion to open surgery (11.5% vs. 0.3% in Groups A and B, respectively). The reason for conversion was the inability to perform sufficient repair laparoscopically in six (11.5%) of the Group A patients, whereas only one patient (0.3%) in Group B required conversion to open surgery for chest problems associated with anesthesia (Table 3).

**Table 3:** Intra-operative findings in the study groups

	Group (A) (*n* = 52)	Group (B) (*n* = 360)	*P* value
Blood loss	200 (100–250)	50 (20–100)	< 0.001*
Operative time	211.92 ± 44.02	111.89 ± 18.86	< 0.001*
Muscle thickness			
Thick	22 (42.3%)	174 (48.3%)	0.416
Thin	30 (57.7%)	186 (51.7%)	
Operative experience			
< 5 cases	14 (26.9%)	27 (7.5%)	< 0.001*
5–10 cases	11 (21.2%)	70 (19.4%)	
10–15 cases	8 (15.4%)	112 (31.1%)	
> 15 cases	19 (36.5%)	151 (41.9%)	
Dissection method			
Harmonic scalpel	31 (59.6%)	216 (60%)	0.692
Harmonic and diathermy	5 (9.6%)	25 (6.9%)	
Ligasure	14 (26.9%)	216 (31.1%)	
Ligasure and diathermy	2 (3.8%)	25 (1.9%)	
Conversion to open	6 (11.5%)	1 (0.3%)	< 0.001*

### Time of detection of mucosal perforation

Most cases of mucosal perforation were detected intraoperatively during LHM (94.2%), although three cases (5.8%) were detected 14–18 days postoperatively (5.8%). We attribute these three cases of delayed detection of mucosal injury to a missed thermal injury during the primary procedure. Most of the perforations in Group A were on the gastric side (63.5%), with the remaining perforations on the esophageal side. The cause of these perforations was either mechanical (71.2%) or thermal (28.8%). The mucosal perforations ranged from 0.5 to 2 cm in length. All the mucosal perforation were repaired by laparoscopic suturing, apart from those in the six patients who needed conversion to open surgery. The three patients with mucosal perforation discovered late underwent surgical repair late postoperatively.

### Post-operative outcomes

Group A showed a significant delay in oral intake and had a longer duration of hospitalization than Group B (*p* < 0.001). A post-operative subphrenic collection occurred in one patient from Group A (1.9%) but none from Group B. This patient was managed by tube drainage and antibiotics. Three patients (5.8%) from Group A required re-operation for late perforation.

### Improvement of symptoms and patient satisfaction

There were no significant differences in any post-operative symptom scores between the two study groups. Treatment failed in 19.2% and 13.3% of the patients in Groups A and B, respectively (*p* = 0.253). Mucosal perforation was associated with a marked decline in post-operative patient satisfaction (*p* = 0.038).

### Follow up

The median duration of follow-up was 57 months for Group A and 49 months for Group B. The incidences of post-operative heartburn, residual dysphagia, and reflux esophagitis were also comparable between the groups. Residual dysphagia was managed initially by endoscopic balloon dilatation, but surgical redo-myotomy was needed in 7.7% and 3.3% of the Group A and B patients, respectively (Table 4). Patients with post-operative GERD symptoms or reflux esophagitis were managed conservatively. Surgical interventions such as redo fundoplication and repair of hiatal hernia were not required in either group.

**Table 4:** Post-operative and follow-up outcomes in the two groups

	Group A (*n* = 52)	Group B (*n* = 360)	*P* value
Abdominal Collection	1 (1.9%)	0 (0%)	0.008*
Re-operation	3 (5.8%)	0 (0%)	< 0.001*
Hospital stay	8 (6–20)	2 (2–2)	< 0.001*
First day oral	5 (4–10)	1 (1–1)	< 0.001*
Follow up period	57 (12–192)	49 (12–190)	0.966
Post dysphagia score	1 (0–2)	0 (0–2)	0.321
Post-chest pain score	0 (0–2)	0 (0–2)	0.549
Post regurgitation score	0 (0–1)	0 (0–1)	0.252
Total post-operative symptoms score	1 (0–3)	1 (0–3)	0.194
Treatment failure	10 (19.2%)	48 (13.3%)	0.253
Heartburn	12 (23%)	56 (15.5%)	0.172
Reflux esophagitis	12 (23%)	52 (14.4%)	0.108
Reflux esophagitis grade			
Grade (A)	(6) (50%)	(34) (65.3%)	0.216
Grade (B)	(6) (50%)	(18) (34.7%)	
Residual dysphagia			
6 months	0 (0%)	0 (0%)	1
12 months	5 (9.6%)	31 (8.6%)	0.811
24 months	0 (0%)	7 (1.9%)	0.310
36 months	1 (1.9%)	2 (0.6%)	0.278
48 months	4 (7.7%)	9 (2.5%)	0.055
Overall residual dysphagia	10 (19.2%)	48 (13.3%)	0.253
Management by balloon dilatation	10 (19.2%)	48 (13.3%)	0.253
Redo myotomy	4 (7.7%)	12 (3.3%)	0.128
Patient satisfaction			
Very satisfied	31 (59.6%)	274 (76.1%)	0.038*
Satisfied	19 (36.5%)	76 (21.1%)	
Unsatisfied	2 (3.8%)	10 (2.8%)	

Thirty of the 52 patients with mucosal injury had perforation on the gastric side and the remaining had esophageal perforation. On assessing the impact of the perforation site on post-operative outcomes, the incidences of heartburn and reflux esophagitis were significantly higher in the patients with gastric perforations (*p* = 0.04), whereas the incidence of residual dysphagia was more commonly associated with esophageal perforation (*p* = 0.001) (Table 5).

**Table 5:** Impact of the site of mucosal injury on outcomes

	Esophageal side (*n* = 22)	Gastric side (*n* = 30)	*P* value
Heart burn	2 (9%)	10 (33.3%)	0.04*
Reflux esophagitis	2 (9%)	10 (33.3%)	0.04*
Reflux esophagitis			
Grade (A)	0 (0%)	6 (60%)	0.154
Grade (B)	2 (100%)	4 (40%)	
Residual dysphagia	9 (40.9%)	1 (3.3%)	0.001*

### Development of a pre-operative risk-scoring system for predicting mucosal injury

The significant risk factors in multivariate analysis were used to form a pre-operative score to assess the risk of perforation before the procedure. The six risk factors were assigned points as follows: age, 1point; BMI ≤ 22 kg/m^2^, 1 point; duration > 48 months, 1 point; albumin < 3.4 gm/dl, 3 points; esophageal diameter ≥ 6 cm, 5 points; and sigmoid esophagus, 5 points. By adding the previous values, this yielded a total score ranging from 0 to 16 (Table 6).

**Table 6:** Pre-operative predictors of mucosal injury

	Multivariate analysis				Points assigned
	OR	95% CI for OR	p	B	
Old age > 45 years	1.03	1.01–1.07	0.020*	0.520	1
BMI ≤ 22 kg/m^2^	0.778	0.709–0.988	0.004*	-0.778	1
Disease duration > 48 m	1.267	1.010–1.436	0.011*	0.820	1
Serum albumin ≤ 3.4 gm/dl	0.866	0.781–0.917	0.013*	-2.885	3
Esophagus diameter ≥ 6 cm	1.393	1.018–1.765	0.005*	5.188	5
Sigmoid shaped type	1.465	1.005–1.802	0.001*	4.640	5

The risk of perforation was classified into three categories according to the total score: low risk (score 0–2), intermediate risk (score 3 -10), and high risk (score 11–16) risk (Table 7).The scoring system showed a satisfactory discriminatory performance (area under the ROC curve, 0.864; 95% CI 0.827–0.895). At score > 1, the sensitivity was 88.5%, the specificity was 70, and the Brier score was 0.084 (95% CI 0.0650–0.102).

**Table 7:** Estimated risk of mucosal perforation

	Total no. of risk factors	Estimated risk (%)	Observed no. of mucosal perforations	
		%	*n*	%
Low risk (estimated risk < 10%)	0	5.3	3	5.8
	1	6.6	3	5.8
	2	8.3	7	13.5
Intermediate risk (estimated risk 10%-50%)	3	10.4	2	3.8
	4	12.9	1	1.9
	5	16.1	1	1.9
	6	19.9	0	0.0
	10	43.0	0	0.0
High risk (estimated risk > 50%)	11	50.9	1	1.9
	12	59.5	5	9.6
	13	68.6	19	36.5
	14	78.1	2	3.8
	15	87.6	3	5.8
	16	96.9	5	9.6

## Discussion

This study was performed to assess the incidence, risk factors, and outcomes of mucosal perforation during laparoscopic Heller myotomy procedures. This complication occurred in 52 of the 412 patients, representing an incidence of 12.6%. This lies within the reported incidence of mucosal injury in the literature, which ranges from 5 to 33% [18–21].

Both univariate and multivariate analyses in this study identified that old age was a significant risk factor for perforation. This could be explained by the increased tissue fragility with advancing age making it more susceptible to injury, even with subtle trauma. This was reported by Metman et al., who stated that tissue frailty is the main cause of perforation in the elderly population [22]. We also found that the perforation group (Group A) had a significantly lower BMI than the other group (Group B). This low BMI could reflect a state of malnutrition secondary to prolonged dysphagia and inadequate oral intake, which was confirmed by the serum albumin findings, which decreased significantly in association with mucosal perforation. As albumin is crucial for tissue healing and integrity, we recommend adjustment of that parameter in patients with hypoalbuminemia prior to surgery, through IV infusion of albumin, as the enteral route is already compromised by the achalasia. Contrarily, another previous study noted no significant difference in BMI between their perforation and non-perforation groups, with mean values of 20.1 kg/m^2^ and 20.7 kg/m^2^, respectively (*p* = 0.179) [11].

We found that long disease duration was a strong predictor of perforation on multivariate analysis. Of course, the increased duration will be associated with a worse nutritional state and wider esophagus, which are risks for the same complication. In the same context, other authors reported that a disease duration of more than 10 years was associated with an increased risk of mucosal perforation (*p* = 0.021) [11].

In our study, the score and severity of dysphagia and chest pain were comparable in the two groups, but both the regurgitation score and severity increased in association with perforation. The total pre-operative symptom score was significantly higher in Group A, which could be explained by the increased prevalence of sigmoid esophagus in the perforation group, which is strongly associated with regurgitation.

Our findings showed that pre-operative endoscopic balloon dilatation was a significant risk factor for mucosal perforation on univariate analysis. Previous balloon dilatation induces submucosal microhemorrhagic areas that heal by fibrosis, which may hinder the correct surgical plane leading to perforation [23]. Likewise, Smith et al. noted a significant increase in the incidence of mucosal perforation in patients who had undergone previous endoscopic intervention (9.7% in Group A vs. 3.6% in Group B; *p* < 0.05) [21].

In the current study, a sigmoid-shaped esophagus and an esophageal transverse diameter of more than 6 cm were strong predictors of mucosal perforation. We think that tortuosity of the esophagus makes the operation more difficult, as separating the longitudinal muscle layer would be more problematic if the tube was not straight. This would also make separation of the muscle from the underlying mucosa more difficult. In a previous similar study, the esophageal shape had a significant impact on operative outcomes, as a sigmoid-shaped esophagus was strongly associated with perforation (0.048). However, the same study failed to show any significant impact of dilatation grade on operative outcomes (*p* = 0.336) [11].

The manometric findings in the current study identified that increased LES pressure and decreased intraabdominal esophageal length were risk factors for perforation. The reason for these findings is still a matter of debate; however, the incidence of fibrosis with long-standing achalasia could explain both the increased LES pressure and its shortening, as any fibrous tissue can be associated with tissue contracture. Surgical experience of fewer than five cases was also identified as a risk factor for mucosal perforation on univariate analysis. This confirms the role of surgical expertise in the prevention of complications and highlights the importance of the learning curve in these operations, which seems to be complete after 16 cases [24]. Similarly, limited surgical experience was associated with an increased risk of mucosal perforation in the study conducted by Tsuboi and colleagues, especially among surgeons with experience of fewer than five cases [11].

We used the significant predictors of mucosal injury identified on the multivariate analysis to create a score to help surgeons predict this serious complication pre-operatively. There is deficiency in the current literature to create a tool to predict mucosal perforation during the Heller procedure, but having such a tool would help us decrease risk by changing the modifiable risk factors, such as surgeon experience. More studies should be conducted to create a universal predictive score for mucosal perforation.

We noted a significant prolongation of the operative time in association with perforation, in accordance with the findings of other studies [11, 13].The increased operative time could be a cause and a result of mucosal perforation at the same time. Of course, perforation takes time to repair and can reflect the degree of operative difficulty, again highlighting that limited surgical experience increases the mucosal perforation risk.

The same concept of operative time could also be applied to blood loss. Mucosal perforation may occur secondary to increased intra-operative bleeding, which obscures the operative field. At the same time, the perforation will need extra surgical manipulation and operative time, which will in turn increase blood loss.

It is noteworthy that not all mucosal perforations were diagnosed and managed in the same setting of the primary procedure. There were three cases of delayed presentation, which required open-approach management. We think that these cases were caused by either a thermal injury that resulted in necrosis and subsequent delayed perforation, or by microscopic tears, which expanded during the post-operative period. We recommend that patients are allowed clear fluids in the first 2 days postoperatively, with semisolid or blended food introduced 1 month after the operation. We asked these patients with delayed perforation about the ingestion of solid or sharp food, and they denied its intake which supports our theory regarding thermal or microscopic injuries. Another study confirmed our findings, as one out of the six cases of mucosal perforation was not recognized during the first surgical procedure [12]. Conversely, other authors reported that all perforations were identified immediately and repaired during the same primary surgical setting [25]. Our findings showed a significant delay in oral intake in the mucosal perforation group, but we allowed time for the mucosal tear to heal before oral intake was reintroduced. Patti et al. also noted a significant delay in oral intake after mucosal perforation [26].

The post-operative symptoms and total scores did not differ between our two groups. In a previous similar study, the severity and frequency of achalasia symptoms subsided after the operation, irrespective of mucosal perforation, with no significant difference between the two groups [11], in accordance with our findings. In the current study, the incidence of post-operative failure was statistically comparable in the two groups. In line with our findings, Salvador et al. also reported that the incidence of mucosal perforation was not associated with an increased post-operative failure rate (*p* = 0.34), as it was encountered in 16% and 10.8% of cases in their perforation and non-perforation groups, respectively [13]. Post-operative heartburn was experienced by 15.4% and 10.6% of patients in Groups A and B, respectively, which was comparable. This is in accordance with a previous report of reflux in 12% and 8% of patients in the perforation and non-perforation groups (*p* = 0.163) [11].

Regarding the location of mucosal perforation, we noted a higher incidence of perforation on the gastric side. Based on our experience, we think that the hypertrophied muscle layer of the esophagus makes dissection and identification of the mucosa easier than on the gastric side. We noted an increase in the incidence of reflux manifestations in patients with gastric perforations, whereas patients with esophageal perforation had higher incidence of residual dysphagia. There is a reasonable explanation for this. We usually start myotomy on the esophageal side, continuing toward the gastric side. We think that the esophageal perforation might distract the focus of the surgeon, who will be paying attention to repair of the perforation before continuing the myotomy. This, in turn, would increase the risk of incomplete myotomy and subsequent residual dysphagia. Conversely, the incidence of perforation on the gastric side may be an indicator for complete or near complete myotomy as the surgeon has already completed the gastric side. The complete myotomy, in turn, will increase the risk of post-operative reflux symptoms.

Our investigation has some limitations, as it was a retrospective study conducted at a single surgical center. Further studies covering the previous drawbacks are needed.

## Conclusion

Many risk factors for mucosal perforation have been identified based on previous data. Correctable parameters like albumin should be corrected prior to surgery, while uncorrectable parameters such as age and a sigmoid esophagus should be managed by experienced surgeons. Following these recommendations will decrease the incidence and negative consequences of this serious complication.

## Data Availability

The data that support the findings of this study are available from the corresponding author, upon reasonable request.
